# Peri-prosthetic infection in the postoperative period of endovascular abdominal aorta aneurysm repair: treatment by percutaneous drainage

**DOI:** 10.31744/einstein_journal/2019RC4668

**Published:** 2019-06-28

**Authors:** Diego Lima Nava Martins, Priscila Mina Falsarella, Antonio Rahal, Rodrigo Gobbo Garcia

**Affiliations:** 1 Hospital Israelita Albert Einstein, São Paulo, SP, Brazil.

**Keywords:** Aortic aneurysm, abdominal, Endovascular procedures, Infection, Drainage/methods, Radiology, interventional, Aneurisma da aorta abdominal, Procedimentos endovasculares, Infecção, Drenagem/métodos, Radiologia intervencionista

## Abstract

Endovascular aneurysm repair is an established technique for treating many infrarenal aortic aneurysms. Infection is one of the most serious complications of this technique, and although percutaneous treatment has been well established for intra-abdominal collections, its use to treat peri-prosthetic fluid collections has not been well determined. In this article we describe a small series of three patients who were treated with percutaneous drainage, with good clinical and imaging responses. Percutaneous drainage is a safe, effective and minimally invasive approach for treating this potentially fatal complication.

## RESUMO

O reparo endovascular de aneurisma é uma técnica para tratamento de diversos aneurismas infrarrenais da aorta. A infecção é uma das complicações mais sérias desse tratamento, e a abordagem percutânea tem sido adotada para coleções intra-abdominais, mas seu uso para tratamento de coleções protéticas vasculares não está bem estabelecido. Descreve-se, neste trabalho, pequena série de três pacientes tratados com drenagem percutânea, que apresentaram boa resposta clínica e de imagem. A drenagem percutânea mostrou-se abordagem segura, efetiva e minimamente invasiva para tratamento desta complicação, que é potencialmente fatal.

## INTRODUCTION

Endovascular aneurysm repair (EVAR) of infrarenal abdominal aortic aneurysm (AAA) has become the standard of care for this condition. Although EVAR is less invasive than conventional surgery, it is not devoid of complications.^(^ ^1^ ^)^ Endoleaks are the most frequent and, although rare (2% incidence), postoperative infection is one of the most serious complications.^(^ ^2^ ^)^ The management traditionally used in this situation is intravenous (IV) antibiotic therapy associated with surgical removal of the infected material, followed by arterial reconstruction.^(^ ^3^ ^,^ ^4^ ^)^ Delayed diagnosis and onset of treatment are associated with higher morbidity with the traditional approach (23 to 75%).^(^ ^2^ ^,^ ^4^ ^)^

The use of primary image-guided percutaneous drainage to treat endograft infections was recently reported, however it is not well established for managing peri-prosthetic fluid collections.^(^ ^5^ ^,^ ^6^ ^)^

The purpose of this study is to describe three cases of successful percutaneous drainage of peri-prosthetic infections in the late postoperative period of EVAR.

## CASE REPORT

### Case 1

**Figure 1:**
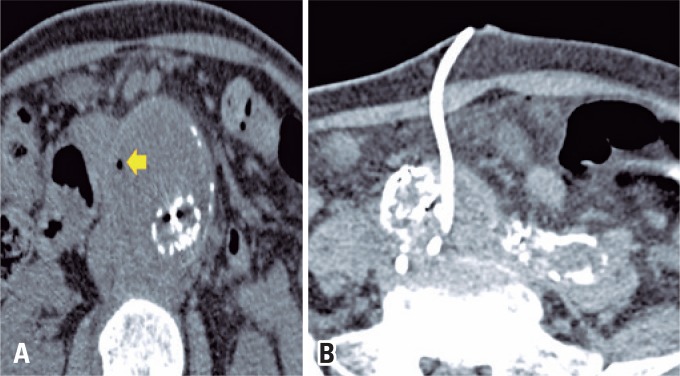
Drainage of peri-prosthetic infected fluid collection. (A) Non-contrast full abdominal CT scan: aneurysm sac with peri-prosthetic fluid collection with gas (arrow); (B) Post-drainage CT: proper placement of the drain, and reduced collection

An 84-year-old male patient evaluated at the emergency department with low back pain, fever and malaise. The patient had past history of endovascular repair of an AAA, 8cm in diameter, with development of a type II endoleak. The abdominal computed tomography (CT) showed a 7cm diameter aneurysm sac, a long-standing hematoma in the retroperitoneum, and presence of peri-prosthetic fluid collection with gas (Figure 1A). Laboratory screening showed leukocytosis and increased inflammatory markers. Due to his high surgical risk, the patient was subjected to ultrasound-guided drainage with an 8.5F pigtail catheter, and 30mL of purulent material was drained. The post-procedure CT scan showed marked reduction in the collection volume (Figure 1B).

Cultures for aerobic and anaerobic microorganisms, and fungi, were negative. The patient had clinical and laboratory improvement, and was discharged in good general condition. Nine months of control CT scans did not show any new peri-prosthetic fluid collections.

### Case 2

**Figure 2:**
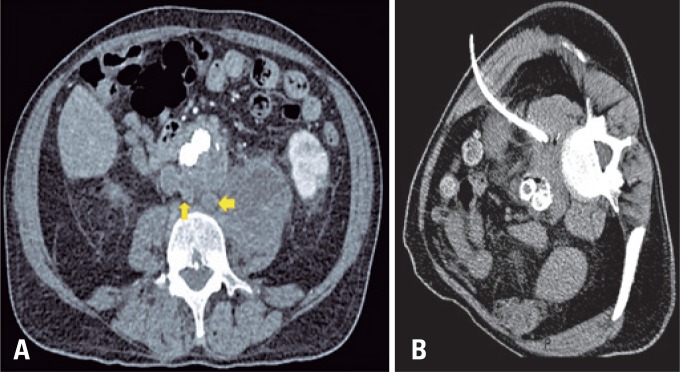
Percutaneous drainage of peri-prosthetic abscess. (A) Computed tomography angiography of the abdomen: aneurysm sac with heterogeneous fluid collection infiltrating the left psoas muscle, and reactive, enlarged retroperitoneal lymph nodes (arrows); (B) Computed tomography -guided drainage with placement of a 12F pigtail catheter

A 68-years old male patient with chronic ruptured infrarenal abdominal aortic aneurysm treated endovascularly 6 years before, currently followed-up with CT angiography of the aorta. The patient had history of coronary artery bypass grafting (CABG), and was assessed at the emergency department with a history of fever and intense low back pain. The abdominal CT showed an enlarged aneurysm sac with heterogeneous peri-aortic infiltration, infiltration of the left psoas muscle, and reactive, enlarged retroperitoneal lymph nodes (Figure 2A). Embolization of the right internal iliac artery was performed, and a new graft was implanted through the common iliac artery, with the distal end in the external iliac artery.

Two weeks later, the patient developed hemodynamic instability, and a new abdominal CT showed increased periaortic fluid collection. The patient underwent CT-guided drainage, with placement of a 12F pigtail catheter (Figure 2B). The patient evolved with clinical improvement and reduction in the abscess volume, from 170mL to 35mL. Cultures were positive for *Salmonella spp*., and the patient was treated with vancomycin, based on the antimicrobial susceptibility testing.

Approximately 75 days later, the patient came back to the emergency department with fever and hemodynamic instability. The abdominal CT showed the abscess had increased in volume, from 35mL to 100mL. A new drainage was performed, with placement of a 12F pigtail catheter, with clinical and laboratory improvement. The patient was discharged with no further symptoms. After 18-month follow-up, no collections were found.

### Case 3

**Figure 3:**
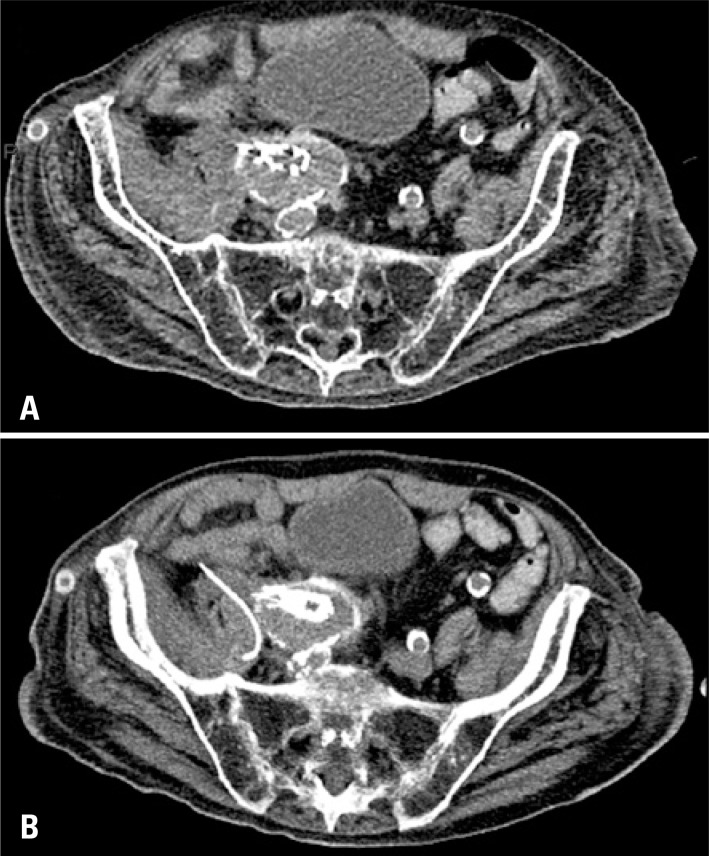
Percutaneous treatment of endograft-related psoas abscess. (A) Non-contrast CT scan confirmed the presence of fluid collection in the right psoas muscle, directly adjacent to the iliac segment of the endograft, and fat densification; (B) Computed tomography -guided percutaneous drainage with placement of a 10F pigtail catheter

A 92-year-old male patient, evaluated at the emergency department with tachycardia and hypotension. The patient had a history of endovascular repair of AAA. The abdominal CT scan showed densification of fat planes adjacent to the right common iliac aneurysm sac, small fluid collections in the right psoas muscle and close to the distal segment of the iliac endograft. (Figure 3A)

The patient was not a proper candidate for open surgical repair and was subjected to CT-guided percutaneous drainage with aspiration of 15mL of purulent secretion and placement of a 10F pigtail catheter (Figure 3B). Cultures showed growth of *Escherichia coli*, and the patient received antibiotic therapy with meropenen, based on the antimicrobial susceptibility testing. Clinical and laboratory parameters improved, and he was discharged with no symptoms after 15 days. After 6-month follow-up, the images showed no recurrence of the collection.

## DISCUSSION

Complications not related to endoleak are relatively infrequent during or after EVAR procedures for AAA. The clinical presentation is usually nonspecific, with fever, strong abdominal or low back pain, and malaise. Laboratory tests showed increased inflammatory markers and leukocytosis. Imaging studies are important for accurate diagnosis, and the presence of collections and gas is the most suggestive finding of peri-prosthetic infection, for which contrast-enhanced CT scans are more sensitive than other imaging modalities.

Therapeutic options are limited, and surgery is the standard treatment.^(^ ^7^ ^)^ However, the potential risks include longer operation times, graft thrombosis, and reinfection.^(^ ^7^ ^)^ In high-risk patients, percutaneous drainage proved to be an excellent alternative, with low morbidity and mortality^(^ ^2^ ^)^ as an adjunct treatment for this condition.

## CONCLUSION

These cases describe a potentially fatal complication of endovascular repair of abdominal aortic aneurysms, and the role of interventional radiology for safe, minimally invasive management of selected patients.
